# NT-proBNP as a Predictive and Prognostic Biomarker for Complications in Hypertensive Pregnancy Disorders

**DOI:** 10.3390/jcm15020519

**Published:** 2026-01-08

**Authors:** Diana Mocuta, Cristina Aur, Ioana Alexandra Zaha, Carmen Delia Nistor Cseppento, Liliana Sachelarie, Anca Huniadi

**Affiliations:** 1 Department of Obstetrics-Gynecology, Faculty of Medicine and Pharmacy, University of Oradea, 1st December Square 10, 410073 Oradea, Romania; dmocuta@uoradea.ro (D.M.); drzahaioana@gmail.com (I.A.Z.); ahuniadi@uoradea.ro (A.H.); 2 Pelican Clinical Hospital, Corneliu Coposu Street 2, 410450 Oradea, Romania; 3 Department of Psycho-Neuroscience and Recovery, Faculty of Medicine and Pharmacy, University of Oradea, 1st December Square 10, 410073 Oradea, Romania; dcseppento@uoradea.ro; 4 Department of Preclinical Discipline, Apollonia University, 700511 Iasi, Romania

**Keywords:** NT-proBNP, sFlt-1/PlGF, preeclampsia (PE), biomarkers, HDP, ROC, NRI

## Abstract

**Background/Objectives:** Hypertensive disorders of pregnancy (HDP) remain a significant cause of maternal and perinatal morbidity worldwide. In some healthcare settings, access to angiogenic testing is limited, underscoring the need for affordable biomarkers to guide risk assessment. NT-proBNP, a marker of myocardial wall stress and cardio-renal dysfunction, may offer complementary prognostic value to the angiogenic sFlt-1/PlGF ratio. **Methods:** In this prospective multicenter observational study, we enrolled 180 pregnant women and categorized them into preeclampsia (PE, *n* = 95), non-PE HDP (gestational or chronic hypertension, *n* = 25), and healthy controls (*n* = 60). NT-proBNP and sFlt-1/PlGF levels were measured at enrollment, after 20 weeks of gestation, predominantly during the second and third trimesters. Associations with proteinuria, uric acid, creatinine, and maternal–fetal complications were examined using multivariable logistic regression adjusted for maternal age, BMI, and gestational age. Discrimination was assessed using receiver operating characteristic (ROC) curve analysis, and the incremental value of NT-proBNP beyond the sFlt-1/PlGF ratio was evaluated using ΔAUC and net reclassification improvement (NRI). **Results:** Median NT-proBNP levels were significantly higher in PE compared with non-PE HDP and controls (*p* < 0.01). NT-proBNP ≥200 pg/mL independently predicted maternal–fetal complications (adjusted OR 3.12, 95% CI 1.41–6.90, *p* = 0.005) and correlated with proteinuria (r = 0.47), creatinine (r = 0.43), and uric acid (r = 0.40) (all *p* < 0.001). sFlt-1/PlGF alone yielded an AUC of 0.84 (95% CI 0.77–0.89), while NT-proBNP alone demonstrated an AUC of 0.78 (0.71–0.84). Combining both biomarkers improved discrimination (AUC 0.88, 95% CI 0.82–0.92), with a ΔAUC of 0.04 (*p* = 0.02) and a continuous NRI of 0.21 (*p* = 0.03). The 200 pg/mL threshold for NT-proBNP achieved 80% sensitivity and 71% specificity (*p* < 0.001). **Conclusions:** NT-proBNP provides independent and complementary prognostic value to the sFlt-1/PlGF ratio in predicting maternal–fetal complications in HDP. A practical threshold of 200 pg/mL aids risk assessment, and integrating NT-proBNP into angiogenic models improves prediction. Further multicenter studies are needed to validate multimarker strategies and their cost-effectiveness.

## 1. Introduction

Hypertensive disorders of pregnancy (HDP) represent one of the most frequent and clinically significant complications of gestation, with substantial consequences for both maternal and perinatal outcomes. Beyond their immediate impact, HDP remains a significant public health concern due to the steadily increasing global burden. Between 1990 and 2021, the worldwide incidence of HDP rose from 31.33 million to 36.10 million cases, representing a relative increase of 15.2%. Meanwhile, prevalence rose from 6.15 million to 36.10 million cases, reflecting a 487% increase over the same period [1]. Currently, HDP complicates 5–10% of all pregnancies worldwide and is responsible for approximately 14% of maternal deaths, according to recent WHO estimates [2]. The burden is particularly pronounced in Eastern Europe, where a higher prevalence of uncontrolled cardiovascular risk factors and limited access to standardized antenatal screening compromise maternal–fetal outcomes [3].

In Romania, HDP remains a significant clinical and public health challenge. However, local research remains limited, particularly regarding biomarkers that may predict disease severity and adverse outcomes. Most studies have focused on conventional laboratory parameters, such as proteinuria or uric acid, while the role of natriuretic peptides, particularly NT-proBNP, has been insufficiently investigated [4]. Generating regional data is essential to complement international findings and to support the use of cost-effective biomarkers in routine practice.

NT-proBNP is a biologically inactive cleavage fragment of proBNP and, unlike BNP, does not bind natriuretic peptide receptors or mediate receptor-dependent signaling. Its clinical relevance lies in its stability in circulation and its role as an integrative biomarker reflecting myocardial wall stress and neurohormonal activation rather than direct biological activity.

Over the past decade, angiogenic markers, particularly soluble fms-like tyrosine kinase-1 (sFlt-1) and placental growth factor (PlGF), have emerged as the most validated predictors of PE, with the sFlt-1/PlGF ratio now incorporated into clinical guidelines in several countries [5]. Despite their strong predictive performance, the widespread use of angiogenic markers is limited by high costs, technical requirements, and reduced availability in many healthcare systems, including those in Eastern Europe. In this context, NT-proBNP is an attractive complementary biomarker, as it reflects cardiovascular and renal strain, can be measured using assays already available in most laboratories, and may provide additional predictive value when combined with angiogenic markers [6,7].

The strong clinical validation of angiogenic biomarkers derives from their direct involvement in the pathophysiology of PE. sFlt-1 acts as a soluble decoy receptor that binds and neutralizes pro-angiogenic factors such as PlGF and VEGF, leading to widespread endothelial dysfunction, impaired placental perfusion, and the systemic manifestations of the disease. Numerous extensive prospective studies and randomized trials have demonstrated that the sFlt-1/PlGF ratio reliably predicts disease onset, severity, and short-term adverse outcomes, resulting in its incorporation into international clinical guidelines for the diagnosis and management of suspected PE.

Against this well-established angiogenic background, comparing NT-proBNP with sFlt-1/PlGF allows evaluation of whether a biomarker reflecting cardiovascular and renal stress can provide complementary prognostic information beyond placental angiogenic imbalance.

The clinical challenge in HDP is the early identification of patients at highest risk for severe maternal complications (stroke, acute renal failure, eclampsia) and adverse fetal outcomes (growth restriction, placental insufficiency, preterm birth). Although advances in obstetric care have improved prognosis, current tools remain insufficient to discriminate which patients will deteriorate. Attention has therefore shifted toward biochemical biomarkers that integrate pathophysiological signals and refine risk stratification.

NT-proBNP, the inactive cleavage product of proBNP secreted by ventricular cardiomyocytes under wall stress, has emerged as a promising candidate. Its levels vary throughout gestation, being higher in the first trimester and decreasing as pregnancy progresses, and are influenced by maternal body mass index and baseline hemodynamics [3,4]. Importantly, elevated NT-proBNP concentrations are consistently reported in PE and severe gestational hypertension, reflecting subclinical cardiac dysfunction [5]. Recent proteomic studies suggest that the natriuretic peptide pathway may play a causal role in HDP pathogenesis, further reinforcing its biological relevance [6]. Clinical studies have also linked high NT-proBNP levels at diagnosis to peripartum cardiomyopathy, postpartum cardiac readmission, and long-term cardiovascular morbidity [7].

Given the study design, the present study aimed to systematically examine the association between NT-proBNP and HDP in a Romanian cohort. We hypothesized that NT-proBNP could provide independent and complementary prognostic information for maternal–fetal complications, adding clinically relevant insight alongside the established sFlt-1/PlGF ratio, rather than serving as a replacement for angiogenic testing. By including both women with HDP and healthy pregnant controls and directly comparing these biomarkers, we aimed to identify clinically relevant NT-proBNP thresholds associated with adverse outcomes and to evaluate whether this marker could serve as a practical, cost-effective option in settings where angiogenic testing is not readily available. Ultimately, we intended to improve risk stratification and help support more timely, individualized care for pregnancies at higher risk.

## 2. Materials and Methods

### 2.1. Study Design and Population

This prospective, multicenter observational study was conducted between November 2024 and October 2025 at the Bihor County Emergency Clinical Hospital and the Pelican Clinical Hospital–Medicover Oradea, Romania, to investigate the prognostic value of NT-proBNP in HDP and its complementary role relative to the angiogenic sFlt-1/PlGF ratio. The study protocol was approved by the Ethics Committee of the Bihor County Emergency Clinical Hospital (No. 35973/21 November 2024), and all procedures were conducted in accordance with the Declaration of Helsinki. Written informed consent was obtained from all participants in accordance with institutional requirements. During the study period, 210 pregnant women were assessed for eligibility; 180 met the inclusion criteria and were included in the final analysis. A total of 180 pregnant women were recruited after 20 weeks’ gestation, provided they had singleton pregnancies, complete clinical and laboratory data, and no conditions known to influence NT-proBNP or angiogenic marker concentrations. Women with multiple pregnancies, pre-existing cardiovascular or renal disease, diabetes mellitus, autoimmune disorders, thyroid dysfunction, or incomplete follow-up were excluded.

For analysis, participants were stratified into three distinct groups. Group A included 95 women diagnosed with PE, among whom both moderate and severe forms were represented. Group B comprised 25 women with HDP without PE, including cases of gestational hypertension and chronic hypertension. Group C consisted of 60 healthy pregnant women with uncomplicated pregnancies, normal blood pressure, and no relevant comorbidities, serving as the control group.

**Figure 1:**
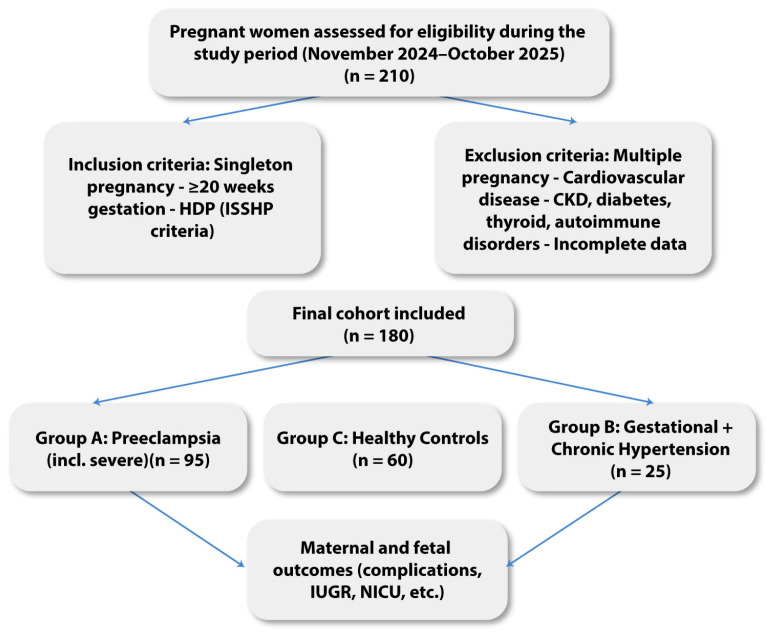
Workflow.

This three-arm design enabled us to compare NT-proBNP and sFlt-1/PlGF levels across pathological and physiological pregnancies, and to assess their individual and combined predictive value for maternal–fetal complications (Figure 1).

### 2.2. Clinical and Laboratory Assessments

Maternal demographic and clinical characteristics were recorded at enrollment, including age, parity, place of residence, educational level, and occupational status. Blood pressure was measured in the seated position after at least five minutes of rest, using a validated automated sphygmomanometer with an appropriately sized cuff applied to the right upper arm. Two consecutive measurements were taken at one-minute intervals, and the mean value was used for analysis.

Biological markers routinely associated with disease severity were assessed in all participants. Proteinuria was quantified from 24 h urine collections and expressed in grams per 24 h. Serum uric acid and creatinine were measured in the central accredited laboratory using standardized automated methods, ensuring inter-assay reproducibility.

For biomarker testing, venous blood samples were collected in EDTA tubes, centrifuged within 30 min, and plasma aliquots were stored at –80 °C until analysis. NT-proBNP was measured at a single time point at study enrollment, after 20 weeks of gestation, predominantly during the second and third trimesters of pregnancy. Gestational age at sampling was recorded for all participants. NT-proBNP concentrations were determined using a commercially available ELISA kit (Human NT-proBNP ELISA Kit, Elabscience Biotechnology Inc., Houston, TX, USA; Catalog No. E-EL-H0185; Lot No. A2024H0523). In parallel, serum levels of sFlt-1 and PlGF were measured using an automated immunoassay platform (Elecsys, Roche Diagnostics, Mannheim, Germany), and the sFlt-1/PlGF ratio was calculated according to the manufacturer’s instructions. All assays were performed in duplicate by laboratory staff who were blinded to participants’ clinical status.

Maternal and fetal outcomes were prospectively recorded throughout the pregnancy and up to delivery. Maternal complications included end-organ dysfunction involving renal, hepatic, neurological, or hematological systems, the occurrence of eclampsia, and indicated preterm delivery before 37 weeks of gestation. Fetal and neonatal outcomes of interest were intrauterine growth restriction (IUGR), stillbirth, a low Apgar score at 5 min, and admission to the neonatal intensive care unit (NICU). These outcomes were used to generate a composite endpoint of adverse maternal–fetal complications for subsequent predictive analyses.

### 2.3. Statistical Analysis

All statistical analyses were performed using SPSS version 27.0 (IBM Corp., Chicago, IL, USA) and MedCalc version 20. Continuous variables were first examined for distribution using the Shapiro–Wilk test. Normally distributed data were expressed as mean ± standard deviation, while skewed variables were reported as median and interquartile range. Categorical variables were presented as absolute numbers and percentages.

Comparisons among the three groups (Group A: PE (PE); Group B: gestational or chronic hypertension; Group C: healthy controls) were performed using the Kruskal–Wallis test, followed, when appropriate, by pairwise Mann–Whitney U tests with the Holm correction. Differences in categorical variables were assessed with the χ^2^ test or Fisher’s exact test.

Correlations between NT-proBNP levels, sFlt-1/PlGF ratio, and classical biochemical markers such as proteinuria, uric acid, and creatinine were evaluated using Spearman’s rank correlation coefficient. Multivariable logistic regression models were then constructed to assess the independent predictive value of NT-proBNP and sFlt-1/PlGF for maternal–fetal complications, adjusting for potential confounders, including maternal age, body mass index, parity, and gestational age at sampling. Effect estimates were expressed as adjusted odds ratios with 95% confidence intervals.

To evaluate discriminatory performance, receiver operating characteristic (ROC) analyses were performed for NT-proBNP, sFlt-1/PlGF, and the combined model including both biomarkers. The area under the curve (AUC) was calculated and compared using the DeLong method. Optimal NT-proBNP thresholds were identified using the Youden index, with particular focus on clinically relevant cut-offs of 150 pg/mL and 200 pg/mL. The incremental predictive value of NT-proBNP beyond the angiogenic ratio was evaluated using changes in AUC (ΔAUC), integrated discrimination improvement (IDI), and continuous net reclassification improvement (NRI). A two-sided *p*-value < 0.05 was considered statistically significant.

## 3. Results

### 3.1. Baseline Characteristics

The demographic and clinical characteristics are shown in Table 1. Women with PE were slightly older, more often from rural areas, and more frequently unemployed than healthy controls, who had higher educational attainment and employment rates (*p* < 0.05).

Following the demographic overview, Table 2 summarizes the incidence of maternal–fetal complications within each study group.

As shown in Table 2, maternal–fetal complications were substantially more frequent in the PE group, providing the clinical context for subsequent biomarker analyses. PE cases exhibited higher rates of severe hypertension, HELLP syndrome, acute kidney injury, and adverse neonatal outcomes. In contrast, these events were uncommon in the non-PE HDP group and rare among healthy controls.

### 3.2. Relationship Between NT-proBNP Levels and HDP

Of the 180 pregnant women included in the study, 95 (52.8%) were classified as Group A (PE), 25 (13.9%) as Group B (gestational or chronic hypertension without PE), and 60 (33.3%) as Group C (healthy controls). Median NT-proBNP levels were highest in Group A, intermediate in Group B, and lowest in Group C, with overall differences between groups statistically significant (*p* < 0.01).

Within the PE subgroup, the interquartile ranges demonstrated considerable variability, particularly among severe cases, suggesting a heterogeneous cardiovascular response to hypertensive stress during pregnancy. Pairwise comparisons confirmed that NT-proBNP concentrations were significantly higher in women with PE than in hypertensive women without PE and healthy controls. No significant difference was observed between Group B and Group C.

These findings indicate that NT-proBNP levels can reliably differentiate PE from both uncomplicated hypertension and normotensive pregnancies; however, they are less effective in distinguishing between gestational and chronic hypertension and healthy states, likely due to smaller sample sizes and milder hemodynamic involvement in these subgroups (Table 3).

### 3.3. NT-proBNP in Relation to Biological Indicators of Disease Severity

NT-proBNP levels showed a positive, statistically significant correlation with proteinuria, uric acid, and serum creatinine, indicating that higher NT-proBNP concentrations were associated with greater disease severity (Table 4).

NT-proBNP showed significant positive correlations with proteinuria, uric acid, and creatinine, indicating its association with renal and metabolic markers of disease severity. In addition, a moderate correlation was observed with the sFlt-1/PlGF ratio (r = 0.42, *p* < 0.001), suggesting that NT-proBNP and angiogenic imbalance reflect complementary but related pathophysiological processes in HDP.

### 3.4. NT-proBNP Levels Across Hypertensive Disorder Subtypes

Median NT-proBNP levels varied across hypertensive disorder subtypes, with the highest values recorded in women with severe PE, followed by moderate PE, gestational hypertension, and chronic hypertension. At the same time, healthy controls consistently displayed the lowest concentrations (Table 5). Overall group differences were statistically significant (*p* < 0.01), primarily driven by the marked elevation observed in severe PE relative to all other categories. Pairwise analyses confirmed that NT-proBNP levels in severe PE were significantly higher than in gestational hypertension, chronic hypertension, and healthy controls (all *p* < 0.01). In contrast, the difference between gestational hypertension and healthy controls was not statistically significant, indicating milder hemodynamic involvement in these cases. Compared with angiogenic markers, NT-proBNP showed a parallel but non-overlapping pattern with the sFlt-1/PlGF ratio. Both biomarkers distinguished severe PE from other groups, but NT-proBNP provided additional information regarding renal and cardiovascular strain. These findings suggest that NT-proBNP may complement sFlt-1/PlGF in risk stratification, particularly in identifying women at higher risk of end-organ dysfunction.

**Figure 2:**
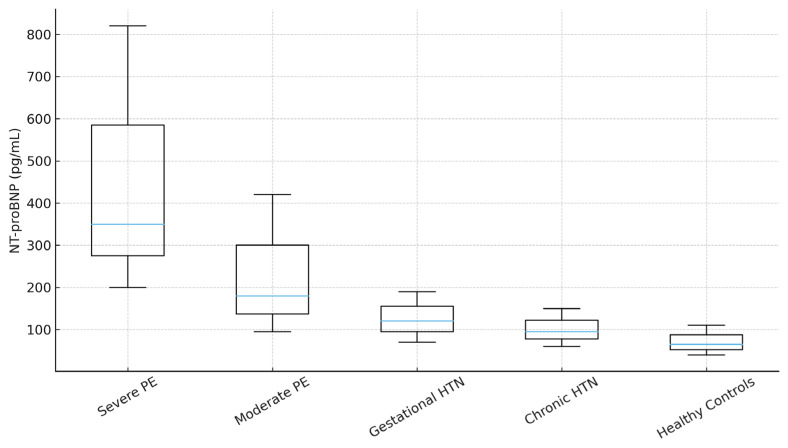
NT-proBNP Levels Across Hypertensive Disorder Subtypes.

Pairwise analyses showed significantly higher NT-proBNP levels in severe PE compared with gestational hypertension, chronic hypertension, and healthy controls (all *p* < 0.01), Figure 2. Differences between gestational hypertension and healthy controls were not statistically significant.

### 3.5. NT-proBNP Threshold for Prediction of Maternal–Fetal Complications

To further explore the predictive role of NT-proBNP in HDP, ROC curve analysis was performed for different thresholds (Table 6). An NT-proBNP value above 200 pg/mL provided the best discrimination for maternal–fetal complications, with an AUC of 0.78 (95% CI, 0.71–0.84), a sensitivity of 80%, and a specificity of 71% (*p* < 0.001). Values above 150 pg/mL have already demonstrated moderate predictive ability. Still, the 200 pg/mL cutoff consistently yielded the strongest performance and was independently associated with a threefold increase in the risk of complications in multivariable logistic regression (adjusted OR, 3.25; 95% CI, 1.42–7.45; *p* = 0.005). When compared with the angiogenic sFlt-1/PlGF ratio, NT-proBNP demonstrated complementary predictive value. The sFlt-1/PlGF ratio alone achieved an AUC of 0.84 (95% CI 0.77–0.89), while NT-proBNP alone reached 0.78. The combination of both markers improved discriminatory performance to an AUC of 0.88 (95% CI, 0.82–0.92), with significant reclassification gains (ΔAUC = 0.04, *p* = 0.02; continuous NRI = 0.21, *p* = 0.03). These results suggest that NT-proBNP not only serves as a cost-effective biomarker but also enhances the prognostic capacity of angiogenic testing, particularly for identifying women at high risk of adverse outcomes.

Table 6 shows that the 200 pg/mL threshold provides the strongest predictive performance for maternal–fetal complications.

## 4. Discussion

NT-proBNP has emerged as a promising biomarker in HDP (HDP), reflecting myocardial wall stress and hemodynamic overload. NT-proBNP is released from ventricular cardiomyocytes in response to increased myocardial wall stress and volume overload and is generally regarded as an integrative marker of cardio–renal strain. In pregnancy, elevated NT-proBNP levels may reflect maladaptive maternal cardiovascular responses, particularly in the setting of increased afterload, endothelial dysfunction, and altered plasma volume regulation that characterize HDP. Angiogenic imbalance, a hallmark of PE, may further amplify myocardial stress through systemic vascular resistance and microvascular dysfunction. From a pathophysiological perspective, natriuretic peptide signaling is therefore thought to integrate hemodynamic, vascular, and renal stress pathways rather than acting as a disease-specific trigger. In this context, NT-proBNP may serve as an integrative biomarker reflecting the complex cardio–vascular–renal interactions underlying HDP severity. Several studies have consistently demonstrated that NT-proBNP levels are elevated in PE compared with normotensive pregnancies, with the highest concentrations observed in severe forms [3,4,8,9,10,11,12,13,14,15,16]. In the present cohort, NT-proBNP values were significantly higher in PE compared with both gestational and chronic hypertension and healthy controls, with severe PE displaying the highest medians. These findings are in line with previous reports describing a progressive increase from gestational hypertension to severe PE, reinforcing the concept that NT-proBNP reflects disease severity [2,10,16,17,18,19]. Beyond blood pressure, NT-proBNP showed significant correlations with proteinuria, uric acid, and serum creatinine, consistent with earlier studies indicating that this biomarker mirrors cardio–renal strain and identifies patients at higher risk of complications [14,16,20]. In our analysis, NT-proBNP also correlated moderately with the sFlt-1/PlGF ratio, supporting the idea that it captures complementary pathophysiological pathways compared to angiogenic markers. ROC curve analysis further demonstrated its predictive potential, with thresholds around 200 pg/mL providing the best balance between sensitivity and specificity for predicting maternal–fetal complications.

These thresholds are also clinically meaningful. Previous studies have shown that NT-proBNP values in the range of 150–200 pg/mL approach the upper limit of normal during late pregnancy and reflect increased hemodynamic stress, subclinical myocardial strain, and early cardio–renal involvement. The 200 pg/mL cut-off, in particular, is consistent with pregnancy-specific reference intervals proposed in population-based cohorts and has repeatedly been associated with a higher risk of severe PE, end-organ dysfunction, and adverse maternal–fetal outcomes. Therefore, the thresholds identified in our ROC analysis are supported not only by statistical performance but also by biological plausibility and clinical evidence. Taken together, these observations strengthen the validity of our findings and align them with previously published data. This performance is consistent with earlier studies reporting AUC values of 0.70–0.75. For instance, Hong et al. demonstrated that BNP predicted adverse maternal outcomes in PE with an AUC of 0.739 [12,21]. At the same time, Nguyen et al. reported higher NT-proBNP levels in severe cases and demonstrated significant predictive ability [2,10,16,17,18]. Moreover, Dockree et al. proposed pregnancy-specific reference intervals, reporting upper limits of approximately 200 pg/mL in early pregnancy and 150 pg/mL in the third trimester, thresholds that align with our findings [8,22].

While associations between NT-proBNP and PE have been previously reported, the present study extends existing evidence by defining a clinically relevant prognostic threshold and by quantifying the incremental value of NT-proBNP beyond angiogenic markers using reclassification analyses.

It should be emphasized that women with PE were enrolled after clinical diagnosis; therefore, the present findings should be interpreted in a prognostic and severity-assessment context rather than as evidence supporting the use of NT-proBNP in PE screening algorithms. Evaluation of NT-proBNP as an early screening biomarker would require prospective first-trimester cohorts and longitudinal follow-up.

Recent investigations have underscored the predictive utility of cut-offs in the 150–200 pg/mL range. Takahashi et al. reported that higher early pregnancy NT-proBNP levels were paradoxically associated with lower subsequent HDP risk, whereas elevated concentrations at diagnosis signaled adverse outcomes [11]. Nan et al. demonstrated that NT-proBNP improved the short-term prediction of PE and placental complications [13]. Meanwhile, Cruz-Lemini et al. showed that combining NT-proBNP with angiogenic markers, such as sFlt-1 and PlGF, improved predictive performance, underscoring the complementary role of NT-proBNP in multimarker models [17,23,24,25,26]. The strengths of this study include the inclusion of a healthy control group, the systematic comparison of NT-proBNP with angiogenic biomarkers, and the identification of a clinically relevant cut-off at 200 pg/mL. However, several limitations must be acknowledged. Although the cohort was larger than in many previous reports, subgroup analyses were still limited by case numbers, particularly for gestational and chronic hypertension. NT-proBNP was measured at a single time point, precluding conclusions about longitudinal changes across pregnancy.

Finally, although angiogenic markers were integrated, further validation in larger multicenter cohorts is required to confirm the generalizability of these findings. Although an ELISA-based approach was used in the present study, the clinical significance of NT-proBNP lies in the biomarker itself, which is widely available in routine clinical practice via automated immunoassay platforms commonly used in cardiology and internal medicine. Our results support its role as a complementary tool to angiogenic markers, particularly in healthcare settings where access to sFlt-1/PlGF testing is limited. Integrating NT-proBNP into clinical algorithms may help refine risk stratification and enable earlier interventions in high-risk pregnancies. Taken together, the evidence indicates that NT-proBNP is consistently elevated in PE, correlates with classical markers of end-organ involvement, and provides clinically relevant predictive ability for maternal–fetal complications. Thresholds around 200 pg/mL appear especially informative, supported by both reference intervals and ROC-based analyses. Nevertheless, heterogeneity between studies and differences in sampling timing highlight the need for further multicenter validation before NT-proBNP can be routinely implemented into diagnostic or prognostic algorithms for HDP. The present study contributes novel data from a Romanian cohort in which NT-proBNP has not been systematically evaluated. The observed correlations with proteinuria, uric acid, creatinine, and angiogenic imbalance reinforce its biological plausibility.

At the same time, identifying a 200 pg/mL cut-off for predicting maternal–fetal complications offers practical value for clinical decision-making. Importantly, these findings align with international evidence indicating that thresholds between 150 and 200 pg/mL predict disease severity and adverse outcomes [11,12,13,16,17,23,24,25,26]. Emerging evidence also suggests that NT-proBNP may act not only as a marker of cardiovascular strain but also as an integrative indicator of maternal adaptation to pregnancy, reflecting the interplay between hemodynamic, renal, and metabolic pathways. Incorporating NT-proBNP into multiparametric models alongside angiogenic and inflammatory biomarkers could enhance early identification of women at risk for severe maternal–fetal complications.

From a clinical perspective, NT-proBNP could offer a practical and accessible tool to support earlier and more nuanced risk stratification in hypertensive pregnancy disorders. Even in settings where angiogenic testing is not readily available, elevated NT-proBNP levels may help clinicians recognize women who require closer surveillance, earlier referral, or more individualized care, ultimately contributing to safer management of high-risk pregnancies.

This study has several limitations. The sample size and between-group imbalance may reduce statistical power, and subgroup analyses are constrained, particularly for the non-PE HDP group, whose distribution reflects the real epidemiological profile of the participating centers. Gestational hypertension and chronic hypertension were combined into a single HDP category following ISSHP and ACOG classifications and in accordance with previous biomarker studies; however, we acknowledge that these entities differ in pathophysiology, and separate analyses were not feasible due to limited subgroup size. Biomarkers were measured at a single time point, precluding longitudinal inferences, and blood sampling occurred within a clinically standardized window after 20 weeks of gestation, which may still introduce residual variability. To mitigate potential gestational age–dependent effects, gestational age at sampling was included as a covariate in all multivariable regression models. Future longitudinal studies with repeated measurements across pregnancy are warranted to further characterize gestational age–related NT-proBNP dynamics. Although angiogenic markers were assessed only in a subset/not available (as applicable), our findings warrant validation in larger multicenter cohorts and head-to-head multimarker models. Another limitation is potential measurement bias arising from laboratory procedures and batch-to-batch variability, despite all samples being processed according to standard protocols.

Additionally, the study design does not allow assessment of NT-proBNP as a screening biomarker for PE, as biomarker measurements were performed after clinical diagnosis of HDP.

## 5. Conclusions

NT-proBNP is consistently elevated in PE, with the highest concentrations observed in severe forms, and correlates with established markers of disease severity, including proteinuria, uric acid, and creatinine. ROC analysis identified thresholds of 150–200 pg/mL as clinically informative, with 200 pg/mL providing the best balance between sensitivity and specificity for predicting maternal–fetal complications. These findings support the role of NT-proBNP as a cost-effective, widely available complementary biomarker that may enhance risk stratification when used in conjunction with angiogenic markers, such as the sFlt-1/PlGF ratio. Nevertheless, larger multicenter studies and longitudinal assessments are needed to validate its integration into routine diagnostic and prognostic algorithms for HDP.

## Figures and Tables

**Table 1:** Demographic and Clinical Characteristics.

Variable	Group A: PE (*n* = 95)	Group B: GH + CH (*n* = 25)	Group C: Healthy Controls (*n* = 60)	*p*-Value
Maternal age (years), mean ± SD	31.8 ± 5.9	30.5 ± 5.4	28.7 ± 4.8	0.021
Place of residence: Rural (%)	55 (57.9%)	16 (64.0%)	24 (40.0%)	0.038
Place of residence: Urban (%)	40 (42.1%)	9 (36.0%)	36 (60.0%)	0.038
Education: Primary/None (%)	20 (21.1%)	6 (24.0%)	4 (6.7%)	0.029
Education: Secondary (%)	18 (18.9%)	5 (20.0%)	10 (16.7%)	0.029
Education: High school (%)	35 (36.8%)	9 (36.0%)	20 (33.3%)	0.029
Education: University (%)	22 (23.2%)	5 (20.0%)	26 (43.3%)	0.029
Occupation: Employed (%)	30 (31.6%)	8 (32.0%)	35 (58.3%)	0.014
Occupation: Unemployed/Jobless (%)	65 (68.4%)	17 (68.0%)	25 (41.7%)	0.014

**Table 2:** Incidence of maternal–fetal complications across study groups.

Complication	Group A: PE (*n* = 95)	Group B: GH + CH (*n* = 25)	Group C: Healthy Controls (*n* = 60)	*p*-Value
Maternal complications				
Severe hypertension (%)	42 (44.2%)	4 (16.0%)	0 (0%)	<0.001
HELLP syndrome (%)	11 (11.6%)	0 (0%)	0 (0%)	0.014
Acute kidney injury (%)	7 (7.4%)	1 (4.0%)	0 (0%)	0.12
Pulmonary edema (%)	5 (5.3%)	0 (0%)	0 (0%)	0.08
Fetal complications				
Preterm birth < 37 w (%)	38 (40.0%)	5 (20.0%)	2 (3.3%)	<0.001
Fetal growth restriction (FGR) (%)	26 (27.4%)	3 (12.0%)	1 (1.7%)	<0.001
Low birth weight < 2500 g (%)	24 (25.3%)	3 (12.0%)	1 (1.7%)	<0.001

**Table 3:** NT-proBNP Levels by Hypertensive Disorder Group.

Group	N (%)	NT-proBNP, Median [IQR] (pg/mL)	*p*-Value
Group A: PE	95 (52.8%)	210.0 [110.5–720.0]	<0.01
Group B: GH + CH (without PE)	25 (13.9%)	120.5 [70.0–190.0]	0.041 vs. A
Group C: Healthy Controls	60 (33.3%)	65.0 [40.0–110.0]	<0.001 vs. A

Note: *p*-values refer to comparisons between Group A (PE) and the combined Groups B (gestational or chronic hypertension) and C (healthy controls). IQR = interquartile range.

**Table 4:** Correlation between NT-proBNP and Biological Markers of Disease Severity.

Marker	Correlation Coefficient (r)	*p*-Value
Proteinuria	0.45	0.002
Uric acid	0.38	0.008
Creatinine	0.41	0.004
sFlt-1/PlGF ratio	0.42	<0.001

**Table 5:** Distribution of NT-proBNP Levels Across Hypertensive Disorders.

Hypertensive Disorder Subtype	NT-proBNP, Median [IQR] (pg/mL)
Severe PE	350.0 [200.0–820.0]
Moderate PE	180.0 [95.0–420.0]
Gestational hypertension	120.5 [70.0–190.0]
Chronic hypertension	95.0 [60.0–150.0]
Healthy controls	65.0 [40.0–110.0]

IQR = interquartile range.

**Table 6:** Predictive Performance of NT-proBNP Thresholds for Maternal–Fetal Complications.

NT-proBNP Cut-Off (pg/mL)	AUC (95% CI)	Sensitivity (%)	Specificity (%)	Adjusted OR (95% CI)	*p*-Value
>100	0.66 (0.58–0.73)	65.0	58.0	1.85 (0.92–3.74)	0.081
>150	0.72 (0.65–0.79)	72.5	64.5	2.42 (1.15–5.12)	0.045
>200	0.78 (0.71–0.84)	80.0	71.0	3.25 (1.42–7.45)	0.005

## Data Availability

The data presented in this study are available from the corresponding author upon reasonable request.

## References

[1] Sun S., Li W., Zhang X., Aziz A.U.R., Zhang N. (2025). Trends in global and regional incidence and prevalence of hypertensive disorders in pregnancy (1990–2021): An age–period–cohort analysis. Sci. Rep..

[2] Countouris M., Mahmoud Z., Cohen J.B., Crousillat D., Hameed A.B., Harrington C.M., Hauspurg A., Honigberg M.C., Lewey J., Lindley K. (2025). Hypertension in Pregnancy and Postpartum: Current Standards and Opportunities to Improve Care. Circulation.

[3] Rosół N., Procyk G., Kacperczyk-Bartnik J., Grabowski M., Gąsecka A. (2024). N-terminal prohormone of brain natriuretic peptide in gestational hypertension and PE—State of the art. Eur. J. Obstet. Gynecol. Reprod. Biol..

[4] Minhas A.S., Rooney M.R., Fang M., Zhang S., Ndumele C.E., Tang O., Schulman S.P., Michos E.D., McEvoy J.W., Echouffo-Tcheugui J. (2023). Prevalence and Correlates of Elevated NT-proBNP in Pregnant Women in the General U.S. Population. JACC Adv..

[5] Iyer N., Gomez J., Rajapreyar I., Boelig R., Al-Kouatly H. (2024). NT-proBNP in PE with severe features as a predictor of adverse maternal cardiac outcomes. Am. J. Obstet. Gynecol..

[6] Schuermans A., Truong B., Ardissino M., Liu Y., Patel R.S., van Dijk D., Kim J., Hernandez A., Roberts J., O’Connor C. (2024). Genetic associations of circulating cardiovascular proteins with gestational hypertension and PE. JAMA Cardiol..

[7] Bacmeister L., Buellesbach A., Glintborg D., Jorgensen J.S., Luef B.M., Birukov A., Heidenreich A., Lindner D., Keller T., Kraeker K. (2025). Third-Trimester NT-proBNP for Pre-eclampsia Risk Prediction: A Comparison With sFlt-1/PlGF in a Population-Based Cohort. JACC Adv..

[8] Dockree S., Brook J., Shine B., James T., Vatish M. (2021). Pregnancy-Specific Reference Intervals for BNP and NT-proBNP—Changes in Natriuretic Peptides Related to Pregnancy. J. Endocr. Soc..

[9] Sarma A.A., Scott N.S. (2023). Dynamics of NT-proBNP in Pregnancy—Why Values May Be Elevated in the First Trimester. JACC Adv..

[10] Suciu V.-E., Leucuța D.-C., Măluțan A.M., Iuhas C., Oancea M., Bucuri C.E., Roman M.P., Ormindean C., Mihu D., Ciortea R. (2025). NT-proBNP and BNP as Biomarkers for Preeclampsia: A Systematic Review and Meta-Analysis. Int. J. Mol. Sci..

[11] Takahashi M., Suzuki L., Takahashi N., Hanaue M., Soda M., Miki T., Tateyama N., Ishihara S., Koshiishi T. (2024). Early-Pregnancy N-Terminal Pro-Brain Natriuretic Peptide Level Is Inversely Associated with HDP Diagnosed after 35 Weeks of Gestation. Sci. Rep..

[12] Bucher V., Mitchell A.R., Gudmundsson P., Atkinson J., Wallin N., Asp J., Sennström M., Hildén K., Edvinsson C., Ek J. (2024). Prediction of Adverse Maternal and Perinatal Outcomes Associated with Pre-Eclampsia and HDP: A Systematic Review and Meta-Analysis. eClinicalMedicine.

[13] Nan M.N., Garrido-Giménez C., García-Osuna A., García Manau P., Ullmo J., Mora J., Sánchez-García O., Platero J., Cruz-Lemini M., Llurba E. (2025). N-Terminal Pro B-Type Natriuretic Peptide as Biomarker to Predict Pre-Eclampsia and Maternal–Fetal Complications. Ultrasound Obstet. Gynecol..

[14] Speksnijder L., Rutten J.H.W., van den Meiracker A.H., de Bruin R.J.A., Lindemans J., Hop W.C.J., Visser W. (2010). Amino-Terminal Pro-Brain Natriuretic Peptide (NT-proBNP) Is a Biomarker of Cardiac Filling Pressures in Pre-Eclampsia. Eur. J. Obstet. Gynecol. Reprod. Biol..

[15] Hauspurg A., Marsh D.J., McNeil R.B., Bairey Merz C.N., Greenland P., Straub A.C., Rouse C.E., Grobman W.A., Pemberton V.L., Silver R.M. (2022). Association of N-Terminal Pro-Brain Natriuretic Peptide Concentration in Early Pregnancy with Development of HDP and Future Hypertension. JAMA Cardiol..

[16] Nguyen T.X., Nguyen V.T., Nguyen-Phan H.N., Hoang B.B. (2022). Serum Levels of NT-ProBNP in Patients with PE. Integr. Blood Press. Control.

[17] Garrido-Giménez C., Cruz-Lemini M., Álvarez F.V., Nan M.N., Carretero F., Fernández-Oliva A., Mora J., Sánchez-García O., García-Osuna Á., Alijotas-Reig J. (2023). Predictive Model for PE Combining sFlt-1, PlGF, NT-proBNP, and Uric Acid as Biomarkers. J. Clin. Med..

[18] Sabattini E., Tinè G., Caricati A., Viscioni L., Cerri S., Radaelli T., Barbieri M., Zamagni G., Stampalija T., Ferrazzi E. (2024). Longitudinal Changes of Systemic Vascular Resistances in Pregnancies Complicated by Hypertensive Disorders and/or Fetal Growth Restriction. J. Obstet. Gynecol..

[19] Rana S., Lemoine E., Granger J.P., Karumanchi S.A. (2019). PE: Pathophysiology, Challenges, and Perspectives. Circ. Res..

[20] Anderson U.D., Olsson M.G., Kristensen K.H., Åkerström B., Hansson S.R. (2018). Biomarkers in PE. Pregnancy Hypertens.

[21] Stepan H., Galindo A., Hund M., Schlembach D., Sillman J., Surbek D., Vatish M. (2023). Clinical Utility of sFlt-1 and PlGF in Screening, Prediction, Diagnosis and Monitoring of Pre-Eclampsia and Fetal Growth Restriction. Ultrasound Obstet. Gynecol..

[22] Zeisler H., Llurba E., Chantraine F., Vatish M., Staff A.C., Sennström M., Olovsson M., Brennecke S.P., Stepan H., Allegranza D. (2016). Predictive Value of the sFlt-1/PlGF Ratio in Women with Suspected PE. N. Engl. J. Med..

[23] Melchiorre K., Sharma R., Thilaganathan B. (2022). Cardiovascular Implications in PE: An Overview. Circulation.

[24] Tomkiewicz J., Darmochwał-Kolarz D.A. (2024). Biomarkers for Early Prediction and Management of PE: A Comprehensive Review. Med. Sci. Monit..

[25] Chaiworapongsa T., Chaemsaithong P., Yeo L., Romero R. (2014). Pre-Eclampsia Part 1: Current Understanding of Its Pathophysiology. Nat. Rev. Nephrol..

[26] Tan M.Y., Syngelaki A., Poon L.C., Rolnik D.L., O’Gorman N., Delgado J.L., Akolekar R., Konstantinidou L., Tsoumpou I., Wright A. (2018). Screening for PE by Maternal Factors and Biomarkers at 11–13 Weeks’ Gestation. Ultrasound Obstet. Gynecol..

